# A cross-sectional analysis of the prevalence of tooth agenesis and structural dental anomalies in association with cleft type in non-syndromic oral cleft patients

**DOI:** 10.1186/s40510-017-0169-x

**Published:** 2017-06-25

**Authors:** Dimitrios Konstantonis, Alexandros Alexandropoulos, Nikoleta Konstantoni, Maria Nassika

**Affiliations:** 1 Department of Orthodontics, School of Dentistry, National and Kapodistrian University of Athens, 2 Thivon st, 115 26 Athens, Greece; 2 Clinic of Orthodontics and Pediatric Dentistry, University of Zurich, Zurich, Switzerland; 3 Center for Advanced Dental Education, Department of Orthodontics, Saint Louis University, Saint Louis, MO USA

**Keywords:** Cleft lip and palate, Dental anomalies, Tooth agenesis, Microdontia

## Abstract

**Background:**

The aim of this study was to investigate the prevalence of tooth agenesis, microdontia, and tooth malformation among non-syndromic oral cleft patients and their potential association with cleft type and gender.

**Methods:**

Intraoral records and radiographs of 154 patients (97 males and 57 females) were examined. The variables assessed were tooth agenesis, microdontia, dental malformations, and cleft types. The statistics included chi-square and Fisher’s exact tests as well as logistic regression to assess any mutual effects of gender and cleft type on the dental variables.

**Results:**

Tooth agenesis occurred in 50% of the sample and microdontia in 18%. Non-statistically significant odds ratios for the association of gender and cleft type with tooth agenesis were obtained. Tooth agenesis was substantially higher at the unilateral right CL + P and the bilateral CL + P in quadrant 1 and at the unilateral left CL + P and bilateral CL + P in quadrant 2. It was also higher, at the isolated cleft palate (CP) in quadrants 3 and 4. These results were attributed to teeth 22 (31.8%) and 12 (21.6%) in the maxilla and to teeth 35 (6.1%) and 45 (5.4%) in the mandible. In unilateral CL + P patients, the cleft quadrant that presented tooth agenesis was associated with the side of the cleft.

**Conclusions:**

Interdisciplinary treatment of the oral cleft patients should take into consideration the high prevalence of tooth agenesis and their association with the different cleft types. The most frequently affected teeth by cleft are by far the upper lateral incisors. Results indicate that tooth agenesis appears to be a genetically controlled anomaly related to the orofacial cleft development through various genetic links and not caused by the cleft disruptive process.

## Background

Cleft lip and palate (CL + P) is the most common craniofacial birth defect in the world [1]. The average prevalence of cleft lip with or without cleft palate is 7.75 and 7.94 per 10,000 live births in the USA and worldwide, respectively, [2].

The most frequent dental anomaly among cleft patients is tooth agenesis [3–7]. The occurrence of tooth agenesis among cleft patients is markedly increased in comparison to the general, non-cleft population [3, 6–11]. Additionally, dental anomalies appear more commonly in the cleft rather than the non-cleft area [4, 10, 12–14]. It is reported that the prevalence of left-sided clefts is higher than right-sided clefts; the cause still remaining unknown [9, 15]. Data from the literature indicate that isolated cleft lip patients (CL) seem to be less affected by dental anomalies outside the cleft area compared to CP or CL + P patients [7]. Furthermore, the permanent dentition seems to be more affected than the primary dentition in patients with unilateral and bilateral CL + P [13].

Other investigations suggest a link between the severity of the cleft type and the number of missing teeth as well as the incidence of dental anomalies [4, 12]. Still, the lateral incisor is reported as the most frequently missing tooth in cleft patients [3, 6, 13, 15, 16]. Also, according to a recent study, the prevalence of lateral incisor agenesis increases in respect to the severity of the cleft [16]. A much higher incidence of agenesis of second premolars was found in the maxilla rather in the mandible in CL + P patients [14, 17]. This agenesis was more frequently observed in the left side and was not gender- or jaw-dependent [9]. Also, contradictory results are reported regarding gender-dependent patterns in the distribution of dental anomalies [4, 18].

It is the aim of this study to identify a contemporary sample of cleft lip and/or palate patients and investigate the prevalence of tooth agenesis and structural dental anomalies and their possible association to the cleft type or gender. Therefore, the null hypothesis of this study was that tooth agenesis and dental structural anomalies are not different between the various types of oral clefts and gender.

## Methods

The data of this study consisted of consecutive cleft patient records obtained from the graduate clinic of the Department of Orthodontics and Pediatric Dentistry of the School of Dentistry of the National and Kapodistrian University in Athens, Greece. An ethics and research committee approval was also obtained (ref. 312/21.09.2016).

Considering that the proportion of patients with tooth agenesis and structural dental anomalies approaches 60%, we found that approximately 160 individuals are needed to ensure that a 99% confidence interval estimate of the proportion is within 10% of the true proportion [19].

By the end of 2016, a total of 154 cleft patient records were thoroughly examined for tooth agenesis and structural dental anomalies. All patients were born between 1977 and 2006 in Greece. Of them, 97 were males and 57 were females. The inclusion criteria were Caucasian male or female non-syndromic patients with complete records including dental casts, photos, and panoramic x-rays; no history of permanent teeth extractions prior to the initial orthodontic screening; and no previous orthodontic treatment received. Third molars were excluded from our assessment. All patient records were taken prior to secondary alveolar bone grafting. Additionally, no pre-surgical orthopedics, gingivoperiosteoplasty or primary bone grafting were performed so that tooth agenesis as well as structural dental anomalies presented in this sample of patients could not be considered iatrogenic. In order to make sure that the agenesis of second premolars was not mistakenly noted due to individual variation, we evaluated all panoramic radiographs of patients older than 8 years of age. All patients received comprehensive orthodontic treatment in the Orthodontic Graduate Clinic.

Since orofacial cleft patients visit the orthodontic department quite early in life, adequate records of intraoral screening and radiographic assessment were readily available. Specifically, the panoramic and cephalometric radiographs along with the patient’s intraoral photographs, dental casts, and charts were minutely examined. In addition, some of the patients’ files contained a cone-beam computed tomography, which was also assessed. In order to assess the intra and inter examiner repeatability, all the patients’ records were reexamined by the principal investigator and by an independent examiner.

The types of orofacial clefts investigated in this research study were isolated cleft lip at the upper right side (CL U R), isolated cleft lip at the upper left side (CL U L), bilateral cleft lip and palate (CL + P B), unilateral cleft lip and palate at the right side (CL + P U R), and unilateral cleft lip and palate at the left side (CL + P U L) and isolated cleft palate (CP). The dental anomalies examined included tooth agenesis, and teeth with morphological discrepancies in regard to mainly their shape and size (malformation and microdontia).

## Statistical analysis

All analyses were performed using the Stata statistical package (Stata/SE 11.0. for Windows; Stata Corporation, College Station, TX, USA). For descriptive purposes, results are presented as frequency and percentages. Chi-square and Fisher’s exact tests were applied to assess comparisons between tooth agenesis, structural dental anomalies, cleft types, and gender. Logistic regression was performed to further assess any mutually adjusted potential effects of gender and cleft type on tooth agenesis and microdontia and to examine potential confounding. Tooth analysis alone or through comparisons of maxillary and mandibular arches was further carried out using Fisher’s exact test. The significance level was predetermined at 5%. To assess intra and inter examiner repeatability, Cohen’s Kappa statistical tests were performed. The reexamination of all 154 cases by the principal and a second investigator resulted in excellent intra and inter examiner agreement.

## Results

### Cleft distribution, tooth agenesis, and structural dental anomalies among patients

**Fig. 1:**
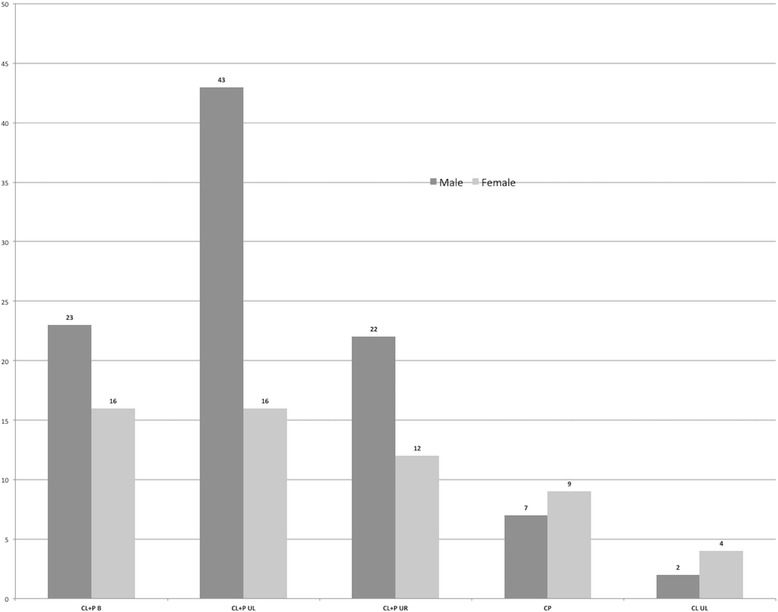
Sample distribution by cleft type and gender. *p* Fisher’s, 0.108

In a total of 154 cleft patients, all cleft types were present except CL U R. The most frequently observed cleft type was CL + P U L (*n* = 59; 38.3%), followed by CL + P B (*n* = 39; 25.3%) and CL + P U R (*n* = 34; 22.1%). On the other hand, the least frequent cleft types were CP (*n* = 16, 10.14%) and CL U L (*n* = 6; 3.9%). A higher incidence of CL + P U L was observed in men (44.3%) compared to women (28.1%), although the overall association between gender and cleft type was not statistically significant (*p* = 0.108) (Fig. 1).

The distribution of tooth agenesis and structural dental anomalies is presented in Table 1 overall and by gender. Of the 154 patients, 77 (50%) presented with tooth agenesis, 28 (18.2%) with microdontia and only one patient with malformation of one tooth. No gender differences occurred overall and for each separate dental anomaly.

**Table 1:** Dental anomalies by gender and overall

	Total 154		Male 97		Female 57		*p* value
Dental anomalies	*N*	%	*N*	%	*N*	%	
Tooth agenesis							0.243^*^
No	77	50.0	45	46.4	32	56.1	
Yes	77	50.0	52	53.6	25	43.9	
Microdontia							0.875^*^
No	126	81.8	79	81.4	47	82.5	
Yes	28	18.2	18	18.6	10	17.5	
Malformation							1.000^**^
No	153	99.3	96	99.0	57	100.0	
Yes	1	0.7	1	1.0	0	0.0	
Overall							0.256^*^
No	56	36.4	32	33.0	24	42.1	
Yes	98	63.6	65	67.0	33	57.9	

^*^From chi-square test; ^**^From Fisher’s exact test

Neither the frequency of tooth agenesis nor microdontia differed between male and female patients. Specifically, 29.9% of the patients had 1 and 14.3% had 2 teeth missing whereas 5.8% of the patients were found to have 3 or more missing teeth. Microdontia was found in 18.2% of the patients. Of these patients, 14.3% presented microdontia in 1 tooth and 3.9% in 2 or more teeth (Table 2).

**Table 2:** Frequency of tooth agenesis and microdontia

	Total		Male		Female		*p* Fisher’s
	154		97		57		
Agenesis freq.	*N*	%	*N*	%	*N*	%	
0	77	50.0	45	46.4	32	56.1	0.255
1	46	29.9	28	28.9	18	31.6	
2	22	14.3	16	16.5	6	10.5	
3+	9	5.8	8	8.2	1	1.8	
Microdontia freq.							0.778
0	126	81.8	79	81.4	47	82.5	
1	22	14.3	15	15.5	7	12.3	
2+	6	3.9	3	3.1	3	5.3	

### Association of tooth agenesis and structural dental anomalies with cleft and gender

**Fig. 2:**
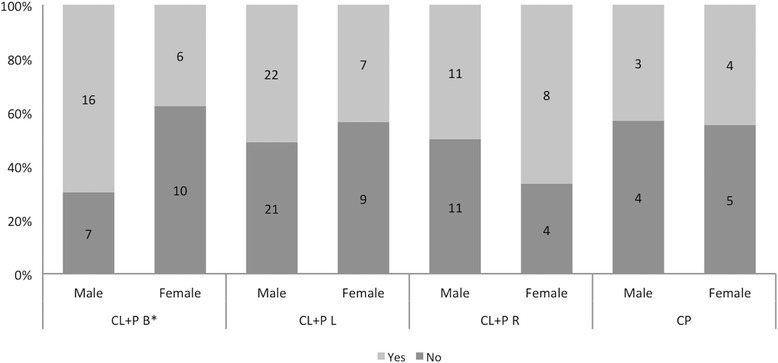
Tooth agenesis by gender and cleft among 148 patients. *Statistically significant difference at 5% level of significance

**Fig. 3:**
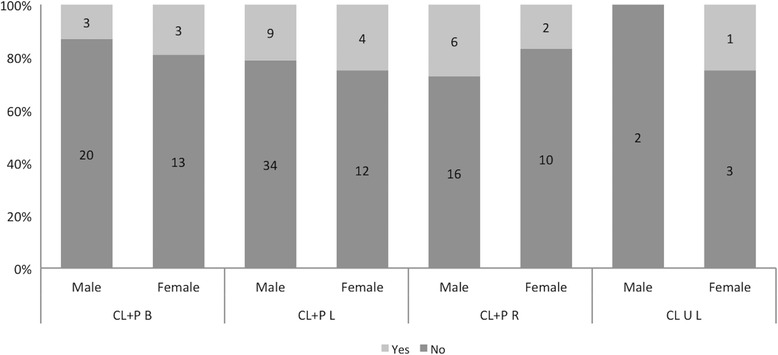
Microdontia by gender and cleft among 138 patients

No tooth agenesis was observed among the 6 patients with CL U L, and no microdontia among the 16 with CP. Still, no differences were found between the different types of clefts in regard to tooth agenesis (*p* = 0.111) and microdontia (*p* = 0.211) (data not shown). After excluding those cleft types with no dental anomalies from the corresponding analyses for tooth agenesis (*N* = 148) and microdontia (*N* = 138), we found that only in the CL + P B group, the male patients were missing significantly more teeth than the female patients (*p* = 0.047) (Figs. 2 and 3). To further explore any potential confounding, we assessed the mutually adjusted effects of gender and cleft type on the occurrence of tooth agenesis or microdontia through logistic regression. Females appeared to have a lower risk for tooth agenesis by 27% compared to men after controlling for cleft type, but the result did not reach statistical significance (OR = 0.73, 95% CI 0.37–1.46). Neither gender nor cleft types were associated with tooth agenesis or structural dental anomalies (*p* values >0.05) (Table 3).

**Table 3:** Odds ratios (95% CI and *p*) for the association of tooth agenesis and microdontia with gender and cleft type (mutually adjusted)

	Tooth agenesis (*N* = 148)				Microdontia (*N* = 138)			
	OR	95% CI		*p*	OR	95% CI		*p*
		Lower	Upper			Lower	Upper	
Gender								
Male	Ref				Ref			
Female	0.73	0.37	1.46	0.375	1.11	0.46	2.70	0.812
Cleft type								
CLP	Ref				Ref			
CLPL	0.71	0.31	1.62	0.422	1.58	0.54	4.62	0.405
CLPR	0.96	0.38	2.44	0.934	1.70	0.52	5.53	0.376
CP	0.63	0.19	2.05	0.441	(Omitted)			
CLL	(Omitted)				1.07	0.10	10.98	0.954

### Tooth agenesis, structural dental anomalies, and cleft type by tooth and by quadrant: inter-quadrant association

The highest prevalence of tooth agenesis occurred for the maxillary left lateral incisor (22), the maxillary right lateral incisor (12), the maxillary right second premolar (15), the mandibular left second premolar (35), and the mandibular right second premolar (45) (Table 4). Both the upper lateral incisors and lower left second premolars were significantly missing in this sample of cleft patients. The upper right lateral incisor was found missing more in the CL + P R group (38.2%); whereas the upper left lateral incisor was primarily missing in the CL + P B group (43.6%) and secondarily in the CL + P L group (40.7%). Furthermore, the lower left second premolar was missing more in the CP group (25%). The CL U L group of patients presented an intact alveolus and no missing teeth or teeth with structural dental anomalies. Microdontia occurred only for the maxillary central and lateral incisors. However, only the upper left lateral incisors presented microdontia, which was significantly larger for the CL + P L group (*p* = 0.028) (Table 5).

**Table 4:** Missing teeth by cleft type in a total of 148 (CL U L patients did not present any missing teeth) cleft-patients

Tooth	*N*	Overall (148)	CL + P B (39)		CL + P L (59)		CL + P R (34)		CP (16)		*p* value^*^
		%	*N*	%	*N*	%	*N*	%	*N*	%	
11	4	2.7	2	5.1	0	0.0	2	5.9	0	0.0	0.185
12	32	*21.6*	11	28.2	6	10.2	13	38.2	2	12.5	*0.007*
13	1	0.7	1	2.6	0	0.0	0	0.0	0	0.0	0.601
14	2	1.4	2	5.1	0	0.0	0	0.0	0	0.0	0.238
15	10	*6.8*	3	7.7	4	6.8	2	5.9	1	6.3	1
16	0										
17	1	0.7	0	0.0	0	0.0	0	0.0	1	6.3	0.108
21	2	1.4	2	5.1	0	0.0	0	0.0	0	0.0	0.238
22	47	*31.8*	17	43.6	24	40.7	3	8.8	3	18.8	*0.001*
23	1	0.7	0	0.0	1	1.7	0	0.0	0	0.0	1
24	4	2.7	3	7.7	1	1.7	0	0.0	0	0.0	0.291
25	6	4.1	3	7.7	1	1.7	1	2.9	1	6.3	0.433
26	0										
27	1	0.7	0	0.0	0	0.0	0	0.0	1	6.3	0.108
31	0										
32	1	0.7	0	0.0	0	0.0	0	0.0	1	6.3	0.108
33	0										
34	0										
35	9	*6.1*	2	5.1	2	3.4	1	2.9	4	25.0	*0.027*
36	0										
37	1	0.7	0	0.0	0	0.0	0	0.0	1	6.3	0.108
41	1	0.7	0	0.0	0	0.0	0	0.0	1	6.3	0.108
42	2	1.4	0	0.0	0	0.0	1	2.9	1	6.3	0.061
43	0										
44	0										
45	8	*5.4*	3	7.7	2	3.4	1	2.9	2	12.5	0.375
46	0										
47	0										

^*^Fisher’s exact test

**Table 5:** Microdontia and cleft type by tooth in a total of 138 cleft patients

Tooth	*N*	Overall (138)	CLL (6)		CLP (39)		CLPL (59)		CLPR (34)		*p* value^*^
		%	*N*	%	*N*	%	*N*	%	*N*	%	
11	4	2.9	0	0.0	2	5.1	0	0.0	2	5.9	0.232
12	13	9.4	0	0.0	5	12.8	2	3.4	6	17.7	*0.091*
21	4	2.9	1	16.7	1	2.6	1	1.7	1	2.9	0.296
22	15	10.9	0	0.0	4	10.3	11	18.6	0	0.0	*0.028*

Table 6 presents the association between tooth agenesis by quadrant and by cleft type. Agenesis of teeth in the upper right quadrant (Q1) occurred mainly in the CL + P R patients (44.1%, *p* = 0.014). Similarly, agenesis of teeth in the upper left quadrant (Q2) occurred mainly in the CL + P B (48.7%) and CL + P L (40.7%) groups of patients (*p* = 0.003). With regard to the mandibular left (Q3) and right (Q4) quadrant, the CP patients were found missing more teeth in these quadrants (*p* = 0.027 and *p* = 0.050, respectively) than any other cleft group.

**Table 6:** Tooth agenesis by quadrant in maxilla and mandible and by cleft type

			CL + P B		CL + P L		CL + P R		CP		*p* value^*^
			*N*	%	*N*	%	*N*	%	*N*	%	
Maxilla	Q1	No	26	66.7	50	84.8	19	55.9	13	81.3	0.014
		Yes	13	33.3	9	15.3	15	44.1	3	18.7	
	Q2	No	20	51.3	35	59.3	30	88.2	12	75.0	0.003
		Yes	19	48.7	24	40.7	4	11.8	4	25.0	
Mandible	Q3	No	37	94.9	57	96.6	33	97.1	12	75.0	0.027
		Yes	2	5.1	2	3.4	1	2.9	4	25.0	
	Q4	No	36	92.3	57	96.6	32	94.1	12	75.0	0.050
		Yes	3	7.7	2	3.4	2	5.9	4	25.0	

^*^Fisher’s exact test

A strong association was found between cleft and non-cleft quadrants in regard to tooth agenesis. More specifically, the association was statistically significant between Q1 and Q2 (*p* = 0.003), Q1 and Q3 (*p* = 0.051), and Q3 and Q4 (*p* < 0.001) (Table 7).

**Table 7:** Tooth agenesis between quadrants

Q1 vs Q2			Maxilla Q2				
			No		Yes		*p*
			*N*	%	*N*	%	*0.003*
	Maxilla Q1	No	84	73.7	30	26.3	
		Yes	19	47.5	21	52.5	
Q1 vs Q3			Mandible Q3				
			No		Yes		*p*
			*N*	%	*N*	%	*0.051*
	Maxilla Q1	No	110	96.5	4	3.5	
		Yes	35	87.5	5	12.5	
Q1 vs Q4			Mandible Q4				
			No		Yes		*p*
			*N*	%	*N*	%	0.155
	Maxilla Q1	No	108	94.7	6	5.3	
		Yes	35	87.5	5	12.5	
Q2 vs Q3			Mandible Q3				
			No		Yes		*p*
			*N*	%	*N*	%	0.480
	Maxilla Q2	No	98	95.2	5	4.8	
		Yes	47	92.2	4	7.8	
Q2 vs Q4			Mandible Q4				
			No		Yes		*p*
			*N*	%	*N*	%	0.507
	Maxilla Q2	No	97	94.2	6	5.8	
		Yes	46	90.2	5	9.8	
Q3 vs Q4			Mandible Q4				
			No		Yes		*p*
			*N*	%	*N*	%	*<0.001*
	Mandible Q3	No	140	96.5	5	3.5	
		Yes	3	33.3	6	66.7	

## Discussion

The distribution of the cleft types between male and female patients did not vary significantly. These results are in concordance with other research reports [14]. Still, the majority of the patients belonged to the CL + P L group (38.3%) in agreement with relative research investigations [9, 15, 20].

In current literature, tooth agenesis is also reported as the most frequent dental anomaly among cleft patients [3–7]. Interestingly, in the CL + P B group, the frequency of tooth agenesis was significantly higher among males. All the other cleft groups showed no differences in the distribution of dental anomalies neither among them nor between genders. Other authors have also reported no differences in dental anomalies between genders [14, 21].

Tooth agenesis in the non-cleft population ranges at considerably smaller numbers than the 50% found in our study. In a cross-sectional study conducted by Lagana [22] in a large sample of 5005 individuals, the prevalence of tooth agenesis was 7,1%, which is in similar range with the reports of Rakhshan [23] (0.15–16.2% excluding the third molars). Lagana [24] also reported that the missing dental units are often the distal teeth in each group of homogeneous teeth: the upper and lower third molars, lateral incisors, and lower second premolars.

A meta-analysis conducted by Polder [25] included 33 studies and investigated the prevalence of non-syndromic tooth agenesis. The results showed that the prevalence of dental agenesis in females was 1.37 times higher than in males. Most individuals were missing one or two permanent teeth, with very few missing more than six. Also, the mandibular second premolar was the most affected tooth, followed by the maxillary lateral incisor and the maxillary second premolar.

The results of our study confirm Dermijian’s reports who postulated that the mechanisms controlling dental development are independent of sexual and somatic maturity thus being influenced by other etiologic factors as clefts [26]. Baek and Kim also reported no differences in the distribution of dental anomalies between Korean male and female patients; whereas Wangsrimongkol et al. examining a sample of 280 Thai patients suggested a gender-dependent pattern in the agenesis of maxillary lateral incisors and maxillary second premolars [4, 18].

In regard to the association of the investigated dental anomalies with the type of cleft, our results showed no differences between the cleft groups coming, thus in contrast with those of Paranaiba et al., where patients with unilateral cleft lip and palate (CLP U) were more frequently affected by dental anomalies than those with bilateral cleft lip and palate (CLP B) [7]. In the same study, CLP U and CLP B were significantly more affected by tooth agenesis than other cleft types. Additionally, Menezes and Vieira reported that in a sample of 146 cleft patients the CL+ P B patients presented more dental anomalies than individuals with incomplete CLP [27]. According to another group of researchers CL + P U had a higher prevalence of tooth agenesis, even in the non-cleft area, in comparison to the normal population [15].

In our sample, the upper lateral incisors followed by the upper right premolar were found missing most frequently in the cleft area. Still, a strong association between the side of the cleft and tooth agenesis was found for the two maxillary quadrants (Q1 and Q2) in CL + P L and in CL + P R patients and for the two mandibular quadrants (Q3 and Q4) in CP patients. In regard to the missing laterals, the findings are in accordance with a study conducted with 203 cleft patients in Brazil stating that agenesis of lateral incisors in CLP U patients was much more frequently noted in the cleft side rather than in the non-cleft side [14]. Still, several research projects conclude that agenesis occurs mainly at the cleft side and the most prevalent missing tooth is the lateral incisor [4, 10, 14–16]. Even in cases of isolated soft tissue cleft lip (the alveolus being intact), dental abnormalities, including tooth agenesis, were significantly more frequent in the cleft side [12]. In agreement with our results, several studies report the maxillary second premolars followed by their mandibular counterparts as the most frequent missing teeth outside the cleft area, thus indicating a genetic link between cleft and tooth agenesis [6, 14, 17].

However, the association between the maxillary right (Q1) and left quadrants (Q2) regarding tooth agenesis was found to be significant. A large percentage (52.5%) of the 40 patients presenting with tooth agenesis in Q1 presented tooth agenesis also in Q2. These results indicate that if a patient presents with tooth agenesis in Q1 is more likely to have agenesis in Q2 compared to a patient who does not present with agenesis in Q1. Still, a strong association was found between Q1 and Q3 indicating that individuals with tooth agenesis at Q1 are more likely to have tooth agenesis also in Q3. To our knowledge in current literature, there are no similar reports examining the association of tooth agenesis quadrants.

The quadrant association findings indicate that tooth agenesis is not directly related to the disruptive osseous defect which occurs at the cleft side but is rather a genetically controlled anomaly related to the orofacial cleft possibly through multifactorial genetic links. These results confirm findings of previous research investigations, which suggest that tooth agenesis encompassing multiple missing teeth has been clearly identified under genetic control of multifactorial inheritance [28–30]. Several of these critical genetic controls assume a mutual part in the development of orofacial clefts [31].

The results of our study will be of valuable help to the clinicians who treat non-syndromic orofacial cleft patients in developing improved interdisciplinary treatment protocols. The high prevalence of tooth agenesis occurred especially in the maxillary arch could be further investigated with the use of the tooth agenesis code (TAC) method.

### Limitations of the study

The limitations of this study can be primarily attributed to the small sample size especially for the groups of cleft lip only (CL U R and CL U L) and cleft palate only (CP). Still, oral clefts comprise a rare disease and collection of large samples can be very challenging. Furthermore, all patients were of Caucasian origin and this constitutes another limitation. In order to achieve an accurate representation of the different cleft types, distribution in non-syndromic oral cleft patients' further investigations should examine different ethnic groups and obtain larger sample sizes.

## Conclusions

The results of this investigation showed that 50% of the oral cleft patients presented with tooth agenesis and 18% with microdontia.

The highest prevalence of tooth agenesis occurred for teeth 22, 12, 15, 35, and 45.

No gender differences were noted overall and for each separate dental anomaly apart from the CL + P B patients where the frequency of tooth agenesis was significantly higher among men.

In CL + P U patients, the cleft quadrant presented tooth agenesis associated with the side of the cleft whereas CP patients showed tooth agenesis in the mandibular arch.

The significant association between quadrants with tooth agenesis and quadrants with no tooth agenesis indicates that tooth agenesis is not directly related to the disruptive osseous defect occuring at the cleft side but is rather a genetically controlled anomaly related to the orofacial cleft process.
